# Effects of multiple doses of gonadotropin-releasing hormone agonist on the luteal-phase support in assisted reproductive cycles: A clinical trial study

**DOI:** 10.18502/ijrm.v19i7.9475

**Published:** 2021-08-16

**Authors:** Maryam Eftekhar, Maryam Mirzaei, Esmat Mangoli, Yasamin Mehrolhasani

**Affiliations:** ^1^Department of Obstetrics and Gynecology, Research and Clinical Center for Infertility, Yazd Reproductive Sciences Institute, Shahid Sadoughi University of Medical Sciences, Yazd, Iran.; ^2^Department of Obstetrics and Gynecology, Jiroft University of Medical Sciences, Jiroft, Kerman, Iran.; ^3^Department of Reproductive Biology, Research and Clinical Center for Infertility, Yazd Reproductive Sciences Institute, Shahid Sadoughi University of Medical Sciences, Yazd, Iran.; ^4^Department of Obstetrics and Gynecology, Bam University of Medical Sciences, Bam, Kerman, Iran.

**Keywords:** Luteal phase, GnRH agonist, ART, Pregnancy rate.

## Abstract

**Background:**

The effect of adding gonadotropin-releasing hormone (GnRH) agonist on the luteal phase support in assisted reproductive technique (ART) cycles is controversial.

**Objective:**

To determine the effects of adding multiple doses of GnRH agonist to the routine luteal phase support on ART cycle outcomes.

**Materials and Methods:**

This clinical trial study included 200 participants who underwent the antagonist protocol at the Research and Clinical Center for Infertility, Yazd, Iran, between January and March 2020. Of the 200, 168 cases who met the inclusion criteria were equally divided into two groups – the case and the control groups. Both groups received progesterone in the luteal phase, following which the case group received GnRH agonist subcutaneously (0/1 mg triptorelin) zero, three, and six days after the fresh embryo transfer, while the control group did not receive anything. Finally, chemical and clinical pregnancy rates, number of mature oocytes, fertilization rate, total dose of gonadotropin, and the estradiol level were determined.

**Results:**

The baseline characteristics were similar in both groups. No significant difference was observed between embryo transfer cycles. Clinical results showed that differences between the fertilization rate, chemical and clinical pregnancies were not significant.

**Conclusion:**

The results showed that receiving multiple doses of GnRH agonist in the luteal phase of ART cycles neither improves embryo implantation nor the pregnancy rates; therefore, further studies are required.

## 1. Introduction 

In a normal menstrual cycle, follicular maturation and ovulation is followed by corpus luteum. It is responsible for the progesterone production, is essential for the endometrium growth, formation of endometrial receptivity, successful implantation, and finally the maintenance of early pregnancy (1).

In a normal reproductive cycle, the luteal phase is formed by the stimulation of the corpus luteum by pituitary luteinizing hormone (LH). However, in assisted reproductive technique (ART) cycles, the luteal phase is defective because the presence of a considerable number of corpora lutea leads to its secretion (2).

It has been reported that luteal-phase defect in the ART cycles cause a decrease in the granular cells by follicular aspiration, inhibition of LH release by the negative feedback of the hypothalamic-pituitary axis, and seeks to increase steroids as well as the suppression of the LH release by analogs (agonists and antagonists) of gonadotrophin-releasing hormone (GnRH) (3).

Controlled ovarian stimulation can accelerate the rate of endometrial maturation, prohibiting both endometrial receptivity and embryo implantation.

Besides, defects in ART cycles are a major concern. The luteal phase support (LPS) in ART is generally performed by the administration of human chorionic gonadotropin (HCG), progesterone, and occasionally estradiol (E2) (4).

Recently, effective use of GnRH agonists for LPS has been reported in both subcutaneous and intrauterine routes. GnRH agonists are effective for LPS, perhaps because in certain doses it shows stimulatory features on corpus luteum, that is, it stimulates LH secretion from the hypophysis and activates local GnRH receptors in the endometrium. However, in some studies, adverse results have been reported regarding the positive effects of using GnRH for LPS (5–8).

The objective of the present work was to investigate the effect of administrating different doses of GnRH agonist to normal LPS on both implantation and pregnancy rate.

## 2. Materials and Methods

### Participants 

**Figure 1:**
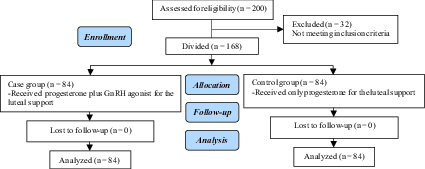
Consort flow diagram.

In this clinical trial, 200 women aged 20–39 yr who visited the Research and Clinical Center Yazd, Iran and underwent ART between January and March 2020 were enrolled (Figure 1). In 32 patients, due to hyper stimulation, a trigger with HCG was not done. These cases were excluded from the study and triggered with GnRH agonist. Other participants (n = 168) were divided into two groups (n = 84/each), both receiving antagonist protocol. While in the case group, in the luteal phase, all women received progesterone plus GnRH agonist, the control group received only progesterone. This study was neither randomized nor blind. The inclusion criterion was only the HCG-triggered cycles that had fresh embryo transfer. However, on the other hand, women with poor embryo quality, ovarian hyperstimulation syndrome (either with previous hyperstimulation syndrome experience, having > 18 follicles >14 mm on the triggering day, or E2 level in the trigger day > 4,000 pmol/l), and severe male factor were excluded.

### Controlled ovarian hyperstimulation and laboratory procedures

After an applied stimulation protocol of 150–225 units of Cinnal-f (CinnaGen, Iran) was administrated subcutaneously from the second day of the cycle, daily subcutaneous administration of 0.25-mg Cetrotide (Merck, Serono, Germany) was started after the dominant follicle size reached 12–13 mm. As the final triggering stage, after at least two-three follicles reached a size of 17 mm, intramuscular administration of HCG (Pregnyl, Merck, Germany) was performed. On the day of HCG injecting, the serum E2 level and endometrial thickness were measured. Oocyte retrieval was performed 34–36 hr after the HCG administration, followed by conventional in vitro fertilization (IVF) and/or intracytoplasmic sperm injection (ICSI).

Embryo grading was done according to the age of the embryos, blastomeres size, and fragmentation (Grades A, B, C) (2). The best embryos were selected and the transfer was carried out utilizing a Labotect catheter (Labotect, GmbH, Rosdorf, Germany) on day 2 or 3 after the oocyte retrieval. Progesterone suppositories (CyclogestⓇ, 400 mg) were used vaginally for LPS in both groups, twice daily from the oocyte-retrieval day until the fetal heart-activity detection. In the case group, in addition to the progesterone suppository, all women received GnRH agonist subcutaneously (0/1 mg triptorelin) zero, three, and six days after the fresh embryo transfer (9).

### Clinical outcomes 

The main outcomes were chemical and clinical pregnancy rates. The number of mature oocytes (MII), fertilization rate, total gonadotropin dose, and the E2 level were measured. Women were followed for biochemical pregnancy (β-hCG > 50 IU/L, 14 days after the embryo transfer [ET] in the serum) and clinical pregnancy (diagnosis of one or more gestational sac with a fetal heartbeat on ultrasound after 6 wk) (10).

### Ethical considerations

This trial was approved by the ethics committee of the Research and Clinical Center for Infertility, Shahid Sadoughi University of Medical Sciences, Yazd, Iran (Code: IR.SSU.RSI.REC.1398.043). Besides, the proposal of the study was registered at the Iranian Registry of Clinical Trials (IRCT). In addition, all participants were informed about the normal infertility treatments and IVF processes and gave their written consent.

### Statistical analysis

The results were statistically analyzed using the Statistical Package for the Social Sciences (SPSS software, version 20.0, Chicago, Illinois). We used the Mann–Whitney test for comparison of non-parametric variables and the student's *t* test for parametric data between the groups. Also, Chi-square tests were used to determine the significant differences between the groups; the significance level was set at p < 0.05.

## 3. Results

In total, 168 women were selected to participate in this study. The study groups had similar baseline characteristics (Table I). The groups also shared similar laboratory characteristics like embryo grade, E2 level on the day of HCG administration, and the number of cumulus oophorus complex (COC), metaphase 2 oocytes (MII), and embryos transferred (Table II).

According to the clinical results, there were insignificant differences in the rate of fertilization between the case and control groups (51.28 ± 23.06 vs. 54.29 ± 23.49, respectively) (p = 0.5). We had chemical pregnancy/transfer 34.5% vs. 36.9% between the case and control groups, respectively (p = 0.87). Also, the rates of clinical pregnancy/transfer were 28.6 vs. 34.5, respectively, in the case and control groups (p = 0.25), which was not significant.

**Table 1:** Basal characteristics of participants in the two groups


**Variable**		**Case group (n = 84)**	**Control group (n = 84)**	**p-value**
Female age (yr)**		32.29 ± 5.12	31.78 ± 4.12	0.39**
Duration of infertility (yr)**		5.6 ± 4.23	6.65 ± 4.49	0.08**
Type of infertility*				
	**Primary**	66 (78.6)	69 (82.1)	0.69*
	**Secondary**	18 (21.4)	15 (17.9)	
AMH (ng/ml)**		3.49 ± 2.47	3.7 ± 2.99	0.84**
Infertility etiologies*				
	**Male factor**	18 (21.42)	15 (17.86)	0.46*
	**Ovarian factor**	12 (14.29)	13 (15.48)	
	**Tubal factor**	5 (5.95)	6 (7.14)	
	**Unexplained**	15 (17.86)	18 (21.42)	
	**Mixed**	34 (40.48)	32 (38.10)	
*Data presented as n (%). Chi square, **Data presented as Mean ± SD. Mann–Whitney test. AMH: Anti-Mullerian hormone				

**Table 2:** Cycle characteristics embryo transfer between groups


**Variable**		**Case group (n = 84)**	**Control group (n = 84)**	**p-value**
No. of transferred embryos**		1. 14± 0.38	1.26 ± 0.68	0.07**
Embryo grade*				
	**A**	42 (50)	45 (53.6)	0.35*
	**B**	33 (39.3)	35 (41.7)	
	**C**	9 (10.7)	4 (4.8)	
Estradiol level on the day of HCG (pg/mL)**		1223.3 ± 805.97	1267.9 ± 707.4	0.29**
**COC**		7.98 ± 2.6	8.63 ± 3.8	0.61**
MII oocyte retrieved*		6. 15 ± 2.27	6. 82 ± 3.25	0.33**
**2PN**		3.16 ± 1.84	3.34 ± 1.69	0.28**
Embryo*		3.09 ± 1.88	3.26 ± 1.75	0.53**
*Data presented as n (%). Chi square, **Data presented as Mean ± SD. Mann-Whitney test. COC: Cumulus oophorus complex, MII: Metaphase 2 oocytes, HCG: Human chorionic gonadotropin				

## 4. Discussion

The study was targeted at evaluating the ART outcomes of adding multiple doses of GnRH agonist for LPS in antagonist protocol and fresh embryo transfer. Our theory was that adding GnRH agonists would support the corpus luteum in the GnRH antagonist protocol and could increase the pregnancy outcome after ART. However, we could not confirm our hypothesis. The outcome of pregnancy was not improved after the addition of GnRH agonists in the luteal phase. The findings of the study disagreed with the results of a meta-analysis (2020). They showed receiving GnRH agonist for LPS on the fifth and sixth day after IVF had a higher ongoing pregnancy rate (11). Furthermore, in another meta-analysis evaluating 13 randomized clinical trials, a significant improvement in live birth rate was witnessed as a result of adding GnRH agonist for luteal support (relative risk [RR] = 1.52; 95% CI 1.20–1.94; p = 0.0006) in comparison with the control (12). Some studies revealed that when a single-dose GnRH agonist was administrated six days after oocyte recovery in antagonist protocol, a considerable increase in the serum concentration of E2 and progesterone on days 7 and 15 after oocyte retrieval, respectively, and beta-HCG 15 days after oocyte retrieval was observed, in contrast to the control. According to their results, a direct effect of GnRH agonist on the embryo and/or endometrium, as well as the increase in both the implantation and clinical pregnancy rates were noticed (13–16). On the other hand, some authors used GnRH agonist for LPS in other protocols (17, 18). In a prospective randomized study carried out by Aboulghar and coworkers, the effect of daily GnRH agonist in the luteal phase was assessed on the IVF and ICSI outcomes in the agonist protocol. Based on their results, the difference in the clinical or ongoing pregnancy rates observed between the groups with or without the continuation of the GnRH agonist in the luteal phase was not statistically significant, confirming no benefit of receiving GnRH-a in the luteal phase of agonist cycles, probably as the GnRH receptor in the endometrium was already saturated, whereas the addition of GnRH agonist to a GnRH antagonist cycles might have different results (17).

In addition, the daily repeated doses of GnRH agonist brought about safe and effective luteal support in the antagonist ART cycles (19). Nevertheless, given the heterogeneity among the trials, administrating GnRH agonist in the luteal phase is still an interesting issue of debate. The results of a retrospective study showed the impact of a mid-luteal-phase GnRH agonist, as an additional LPS, in the cases undergoing ICSI cycles and different subgroups. They reported higher rates of implantation, clinical pregnancy, and live birth for the cases receiving LPS with decapeptyl. Besides, in the analysis carried out on the subgroups, decapeptyl boosted both the clinical pregnancy and live birth rates in participants having basal FSH > 8 mIU/mL, the mature oocytes number of whom was three or fewer (20). Although the administration of GnRH agonists after the transfer of an embryo in ART cycles was initially assumed to be effective on the growth of the embryo and the improvement of embryo developmental potential, further researches confirmed that it might also affect uterine receptivity and corpus luteum function (21). These contradictory results require future studies on the function of GnRH agonists in the luteal phase. One of the limitations of this study was the wide range of participants that may have led to these contradictory results. More studies are suggested to be done in this field and these studies should be carried out considering the participants' age, infertility cause, the quality and quantity of the transferred embryos, the administration timing, the quality of ART protocol, and the duration of GnRH agonist supplementation. Also, it seems necessary to analyze the subgroups to eliminate some confounding variables.

## 5. Conclusion

In conclusion, the results showed the administration of multiple doses of GnRH agonist in the luteal phase of ART cycles did not improve embryo implantation and pregnancy rates and needs further research.

##  Conflict of Interest

The authors have no conflict of interest to declare.
